# Cheap Food in Paris

**Published:** 1864-04

**Authors:** 


*Medical and Surgical Reporter.*

Cheap Food in Paris.—The Philanthropic Society of Paris, has published its report for 1862. From the ten cheap food kitchens established in different parts of the capital, it distributed 290,016 portions of food, the produce of which was 11,709 francs, and the expenses 22,522, francs. In the six dispensaries, 1,775 patients received medical assistance, whilst, 1,659 gratutious consultations were given. Out of that number of sick, 81 died, being a smaller proportion than takes place in the hospitals. The expenses of those dispensaries for 1862, amounted to 41,985 francs, including medicines and baths, or an average of about 24 francs per patient. The budget of the Society for 1862, is stated as follows: The balance in hand was 35,508 francs, and the receipts 66,665 francs, making a total of 102,173 francs. The general expenses amounted to 77,856 francs, showing a surplus of receipts of 24,317 francs. As you are no doubt, aware five francs equal a dollar.—*Medical and Surgical Reporter.*

